# Sirt1 antisense long non-coding RNA attenuates pulmonary fibrosis through sirt1-mediated epithelial-mesenchymal transition

**DOI:** 10.18632/aging.102882

**Published:** 2020-03-06

**Authors:** Weibin Qian, Xinrui Cai, Qiuhai Qian

**Affiliations:** 1 Department of Lung Disease, Affiliated Hospital of Shandong University of Traditional Chinese Medicine, Jinan 250011, Shandong, P.R. China; 2 Department of Traditional Chinese Medicine, Shandong Academy of Occupational Health and Occupational Medicine, Shandong First Medical University and Shandong Academy of Medical Sciences, Jinan 250062, Shandong, P.R. China; 3 Department of Endocrinology, Affiliated Hospital of Shandong University of Traditional Chinese Medicine, Jinan 250011, Shandong, P.R. China

**Keywords:** idiopathic pulmonary fibrosis, epithelial-mesenchymal transition, antisense lncRNA, sirt1, astragaloside IV

## Abstract

Long noncoding RNAs sirt1 antisense (sirt1 AS) was reported to play crucial roles in the progression of organ fibrosis. However, the roles of sirt1 AS in idiopathic pulmonary fibrosis (IPF) are still unknown. In addition, we have previously demonstrated that astragaloside IV (ASV), a bioactive saponin extract of the *Astragalus root*, significantly alleviates IPF by inhibiting transforming growth factor β1 (TGF-β1) induced epithelial-mesenchymal transition (EMT). Further investigations into the influence of ASV on lncRNAs expression will be helpful to delineate the complex regulatory networks underlying the biological function of ASV. Here, we found sirt1 AS expression was significantly decreased in BLM-induced pulmonary fibrosis. We further found that sirt1 AS effectively inhibited TGF-β1-meidated EMT in vitro and alleviated the progression of IPF in vivo. Mechanistically, sirt1 AS was validate to enhance the stability of sirt1 and increased sirt1 expression, thereby to inhibit EMT in IPF. Furthermore, we demonstrated that ASV treatment increased sirt1 AS expression and silencing of sirt1 AS impaired anti-fibrosis effects of ASV on IPF. Collectively, sirt1 AS was critical for ASV-mediated inhibition of IPF progression and targeting of sirt1 AS by ASV could be a potential therapeutic approach for IPF.

## INTRODUCTION

Idiopathic pulmonary fibrosis (IPF) a progressive interstitial pneumonia with poor prognosis [1]. The pathogenesis of IPF is mostly attributed to the fibroblast/myofibroblast trans-differentiation [2] and excessive deposition of extracellular matrix (ECM) components [3]. However, the etiology of IPF is complex [4] and no effective medication is currently available for managing its progression [5]. The pathophysiological mechanisms of its occurrence and development of novel therapeutic strategies is urgently needed.

Long noncoding RNAs (lncRNAs) are a large class of noncoding transcripts that are>200 nt long and that lack protein-coding capacity [6]. Emerging evidence suggests that lncRNAs play an important role in the pathogenesis of IPF [7] and a number of lncRNAs have been identified as the regulators of the epithelial-mesenchymal transition (EMT) in IPF [8, 9]. EMT is a process in which fully differentiated epithelial cells are transformed into a mesenchymal phenotype, with the loss of epithelial markers (such as E-cadherin), and an increase in cell motility associated protein (such as α-SMA), and EMT plays an important role in pulmonary fibrosis [10]. Meanwhile, a subset of lncRNAs is a class of so-called the natural antisense transcripts (NATs), which containing transcripts with sequence complementarity to other RNAs [11]. Many NATs are described as lncRNAs without information about the co-existence of other transcripts from the same genomic origin [12]. Currently, antisense lncRNAs have been reported to regulate cell proliferation and differentiation [13]. Furthermore, we have previously demonstrated that lncRNA zinc finger E-box binding homeobox 1 (ZEB1) antisense RNA1 could facilitate EMT by regulating miR-141-3p/ZEB1 axis in IPF rats [14]. However, there is no more information about the function of antisense lncRNAs in IPF.

Sirt1, a member of sirtuins family, is suggested to regulate the balance between myoblast proliferation and differentiation [15]. Overexpression of sirt1 could significantly attenuate IPF via inhibiting EMT progression [16]. However, the exact mechanisms underlying the function of sirt1 still need to be clarified. We noticed that sirt1 NAT, also known as lncRNA sirt1 antisense (sirt1 AS) could form RNA hybrid double strands with sirt1 mRNA to increase its mRNA stability [17]. And sirt1 AS can regulate cardiomyocyte proliferation and cardiac regeneration by interacting and stabilizing sirt1 mRNA [18]. Due to the role of sirt1 in IPF, we therefore hypothesize that sirt1 AS may also be implicated in the fibrogenesis of IPF. On the other hand, our previous study found that astragaloside IV (ASV), a bioactive saponin extract of the *Astragalus root*, could attenuate IPF by inhibiting TGF-β1-dependent EMT [19] and ASV was also reported to up-regulate sirt1 expression to inhibit glucose-induced EMT of podocytes [20]. We thus speculate that sirt1 may be involved in the protective role of ASV on IPF. Taken together, the first aim of the current study is to reveal the role of sirt1 AS during IPF progression and its potential mechanism. The second is to investigate whether sirt1 AS was involved in the anti-fibrosis of ASV on IPF.

## RESULTS

### Sirt1 AS inhibits TGF-β1-mediated fibrinogenesis

**Figure 1:**
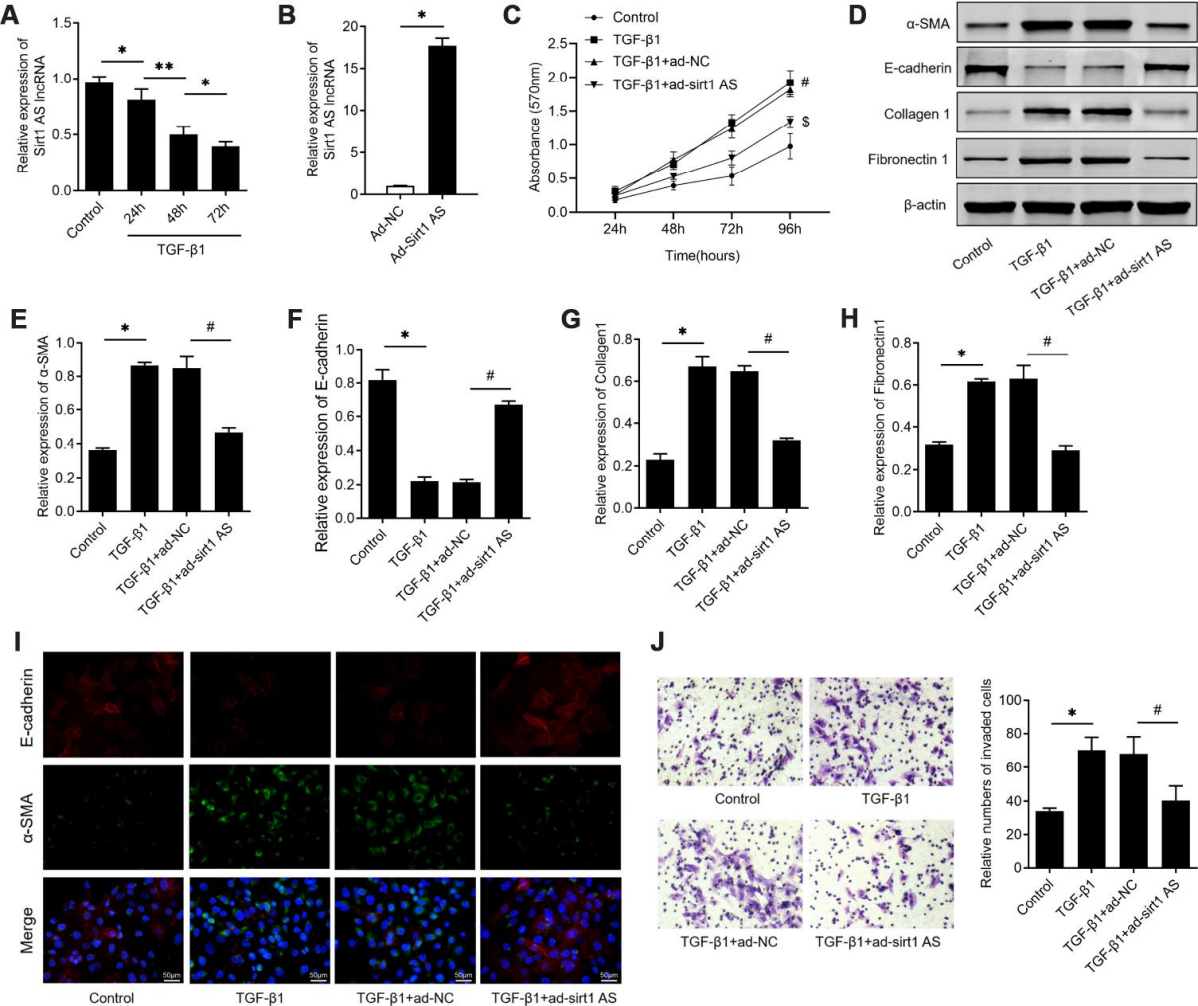
**lncRNA sirt1 antisense (AS) inhibits TGF-β1-induced fibrogenesis in alveolar epithelial cells.** (**A**) Relative expression of sirt1 AS in alveolar epithelial (RLE-6TN) cells with 10 ng/ml TGF-β1 treatment for various time, as indicated. (**B**) Relative expression of sirt1 AS in RLE-6TN cells infected with adenovirus-mediated overexpression sirt1 AS or adenovirus negative control. (**C**) CCK-8 assay was used to detect the cell viability of RLE-6TN cells with or without overexpression of sirt1 AS up TGF-β1 treatment. (**D**–**H**) Relative expression of epithelial-mesenchymal transition (EMT) related markers α-SMA, E-cadherin, collagen1 and fibronectin1 in RLE-6TN cells with or without overexpression of sirt1 AS up TGF-β1 treatment; and densitometry quantified data of above indicated markers, represented as a fold change to β-actin. (**I**) Immunofluorescence staining showing the overlap of α-SMA and E-cadherin in RLE-6TN cells with indicated treatment. (**J**) Transwell assay was performed to investigate the migration ability of RLE-6TN cells with indicated treatment. * P<0.05 vs. control group; # P<0.05 vs. TGF-β1+ad-NC group.

Firstly, we examined the expression levels of sirt1 AS in TGF-β1 treated RLE-6TN cells, with the finding that 10 ng/ml TGF-β1 treatment down-regulated sirt1 AS expression in a time-dependent manner (Figure 1A). We therefore overexpression of sirt1 AS to explore its role in TGF-β1-mediated fibrinogenesis (Figure 1B). As depicted in Figure 1C, 10 ng/ml TGF-β1 treatment resulted in significantly increase in cell viability (Figure 1C). whereas, overexpression of sirt1 AS notably suppressed TGF-β1-induced cell viability (Figure 1D). In addition, the results of western blot in Figure 1E–1H revealed that TGF-β1 treatment induced increase in the protein expression of α-SMA, collagen1 and fibronectin1, and decrease in E-cadherin. However, overexpression of sirt1 AS in TGF-β1-treated RLE-6TN cells could reverse the change of above-mentioned EMT markers. Using immunofluorescence analysis, we confirmed that overexpression of sirt1 AS could reverse TGF-β1-induced EMT, as evidenced by increased E-cadherin and decreased α-SMA expression (Figure 1I). Furthermore, the results of Transwell assay showed that overexpression of sirt1 AS significantly abolished TGF-β1-induced cell migration (Figure 1J). Collectively, these results suggested that overexpression of sirt1 AS suppressed TGF-β1-mediated fibrinogenesis in pulmonary alveolar cell.

### Sirt1 AS alleviates BLM-induced pulmonary fibrosis

**Figure 2:**
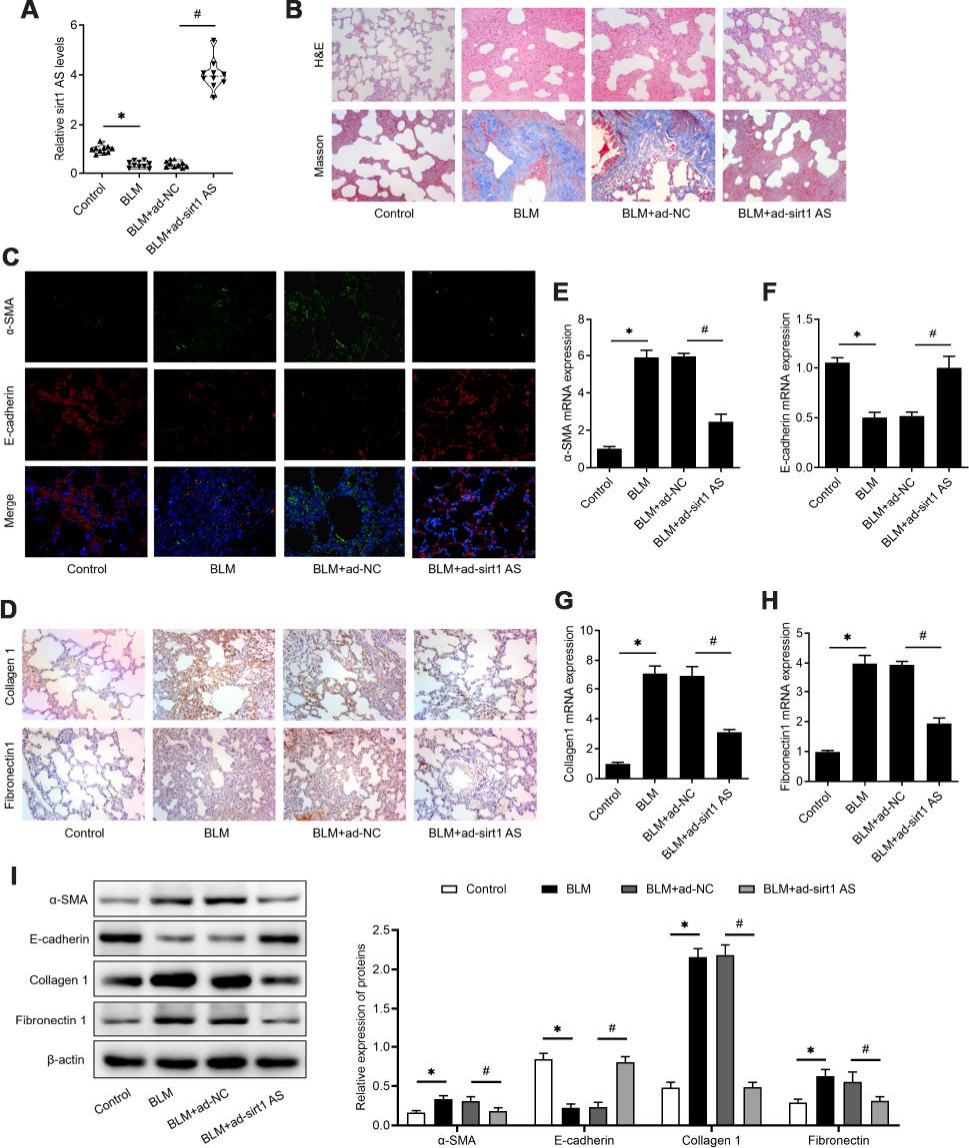
**lncRNA sirt1 antisense (AS) alleviates bleomycin (BLM)-induced pulmonary fibrosis**. (**A**) The expression of sirt1 AS in the pulmonary tissues from control mice or bleomycin (BLM) treated mice that treated with adenovirus sirt1 AS and ad-NC (n=10 per group). (**B**) H&E and Masson’s trichrome staining showed pulmonary injury and collagen deposition of pulmonary tissues in mice with or without sirt1 AS overexpression. (**C**) Immunofluorescence staining showing the overlap of E-cadherin (red) and a-SMA (green) in pulmonary tissues with or without sirt1 AS overexpression. (**D**) Immunohistochemistry analysis indicated the accumulation of collagen 1 and fibronectin 1 in the lung with or without sirt1 AS overexpression. (**E**–**H**) QPCR analysis demonstrated the expression of epithelial-mesenchymal transition (EMT)-related markers, α-SMA, E-cadherin, Collagen 1 and Fibronectin1 mRNA in indicated groups (n=10 per group). (**I**) Western blot showed the relative expression of EMT related markers α-SMA, E-cadherin, collagen1 and fibronectin1 from mice with or without overexpression of sirt1 AS up bleomycin (BLM) treatment; and densitometry quantified data of above indicated markers, represented as a fold change to β-actin. * P<0.05 vs. control group; # P<0.05 vs. BLM+ad-NC group.

To validate the effect of sirt1 AS in IPF, mice were administrated with bleomycin (BLM) to induce IPF. The expression of sirt1 AS was also found to be down-regulated in lung tissues from IPF mice, and its expression was significantly up-regulated in IPF mice transfected with Ad-sirt1 AS (Figure 2A). We found that overexpression of sirt1 AS significantly alleviated BLM-induced lung tissues injury and collagen accumulation (Figure 2B). Through immunofluorescence and IHC analysis, we found overexpression of sirt1 AS could increase E-cadherin protein and decrease α-SMA, collagen1 and fibronectin1 protein in lung tissues (Figure 2C–2D). Accordingly, treated with Ad-sirt1-AS inhibited the up-regulation of α-SMA, collagen1 and fibronectin1 mRNA, and reversed the down-regulation of E-cadherin mRNA in IPF mice (Figure 2E–2H). Furthermore, to quantificationally validate the EMT in IPF mice, western blot was used. The results in Figure 2I showed that overexpression of sirt1-AS notably inhibited the up-regulation of α-SMA, collagen1 and fibronectin1 protein, and reversed the down-regulation of E-cadherin protein in IPF mice. Above data showed that overexpression of sirt1 AS alleviated BLM-mediated IPF through inhibiting EMT.

### Sirt1 AS increases the stability of sirt1 mRNA in alveolar cells

**Figure 3:**
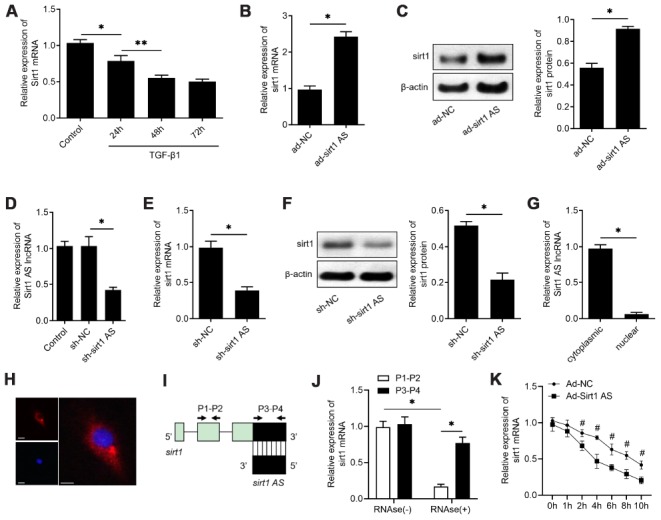
**lncRNA sirt1 antisense (AS) upregulates sirt1 expression by increasing the stability of sirt1 mRNA.** (**A**) Real-time qPCR analysis of sirt1 mRNA levels in RLE-6TN cells treated with 10 ng/ml TGF-β1 treatment for various time, as indicated. (**B**) QPCR of sirt1 mRNA in RLE-6TN cells transfected with ad-sirt1 AS or ad-NC. (**C**) Western blot analysis and quantitative analyses of sirt1 protein levels in RLE-6TN cells transfected with ad-sirt1 AS or ad-NC. (**D**) QPCR was used to validate the knockdown efficiency of lentivirus-mediated sh-sirt1 AS. (**E**) QPCR of sirt1 mRNA in RLE-6TN cells transfected with shRNA against sirt1 AS (sh-sirt1 AS) or sh-NC. (**F**) Western blot assay and quantitative analyses of sirt1 protein levels in RLE-6TN cells transfected with sh-NC or sh-sirt1 AS. (**G**) Nucleocytoplasmic separation results confirmed that sirt1 AS was almost all expressed in the cytoplasm of RLE-6TN cells by using qPCR analysis. (**H**) RNA FISH was used to determine the location of endogenous sirt1 AS (red) expression in RLE-6TN cells. (**I**) Nonoverlapping (P1-P2) and overlapping (P3-P4) primer positions for ribonuclease protection assay (RPA). (**J**) RPA was performed on RNA samples from RLE-6TN cells. (**K**) RNA stability assay of sirt1 mRNA expression in RLE-6TN cells transfected with ad-sirt1 AS or ad-NC. * P<0.05 vs. negative control group.

We then further explore the mechanism how sirt1 AS regulates the IPF progress. A previously studies found sirt1 could control EMT progress of lung fibrosis [21], and a recent study also found sirt1 AS could regulate srit1 expression in cardiomyocytes. Therefore, we performed experiments to determine whether sirt1 was involved in the role of sirt1 AS in IPF. As shown in Figure 3A, 10 ng/ml TGF-β1 down-regulated sirt1 mRNA expression in a time-dependent manner, which was consistent with the change of sirt1 AS in Figure 3A. Overexpression of sirt1 AS significantly increased sirt1 mRNA (Figure 3B) and sirt1 protein expression (Figure 3C). Loss-of-function was then performed by transfected with RLE-6TN cells with lentivirus-mediated shRNA and sh-sirt1 AS significantly reduced sirt1 AS expression, as detected by RT-qPCR assay (Figure 3D). On the contrary, knockdown of sirt1 AS decreased the expression of sirt1 at both mRNA (Figure 3E) and protein (Figure 3F) levels. We then examined the cellular distribution of Sirt1 AS in RLE-6TN cells. Sirt1 AS was mainly located in the cytoplasm (Figure 3G) and subsequent FISH assay validated that Sirt1 AS located in the cytoplasm of RLE-6TN cells (Figure 3H). We used a ribonuclease protection assay on cell RNA to determine the possibility of RNA duplex formation of sirt1 AS and sirt1. Interestingly, overlapping part of both transcripts was protected from degradation (Figure 3I and 3J), suggesting that Sirt1 AS and sirt1 mRNA form an RNA duplex. Moreover, actinomycin D was used to suppress cells transcription, and the loss of sirt1 mRNA expression was detected. Overexpression of sirt1 AS elongated the half-life of Sirt1 mRNA (Figure 3K) in RLE-6TN cells. Taken together, these data indicated that sirt1 AS could increase the stability of sirt1 mRNA and upregulate sirt1 expression in alveolar cells.

### Sirt1 is involved in sirt1 AS regulated fibrogenesis

**Figure 4:**
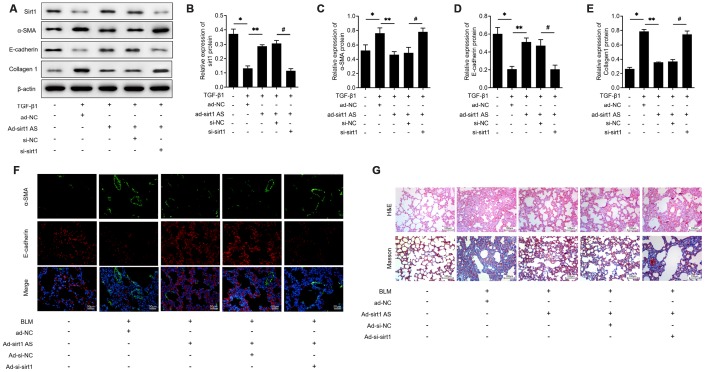
**Sirt1 is involved in the anti-fibrosis function of lncRNA sirt1 antisense (AS).** (**A**–**E**) Western blot assay and quantitative analyses of sirt1 protein levels in RLE-6TN cells transfected with ad-NC, ad-sirt1 AS, ad-sirt1 AS+si-NC, ad-sirt1 AS+si-sirt1 under TGF-β1 treatment. (**F**) Immunofluorescence staining showing the overlap of E-cadherin (red) and α-SMA (green) in pulmonary tissues from mice intratracheally infected with ad-NC, ad-sirt1 AS, ad-sirt1 AS+ad-si-NC, ad-sirt1 AS+ad-si-sirt1. (**G**) H&E and Masson’s trichrome staining showed pulmonary injury and collagen deposition of pulmonary tissues in mice with above indicated treatment. * P<0.05 vs. control; ** P<0.05 vs. TGF-β1+ad-NC; # P<0.05 vs. TGF-β1+ad-sirt1 Ad+si-NC.

On the base of above findings, we therefore investigated whether sirt1 AS influenced the EMT progress in a sirt1 dependent way. The results of western blot analysis in Figure 4A showed that RLE-6TN cells transfected with si-sirt1 significantly down-regulated sirt1 proteins levels (Figure 4B) and knockdown of sirt1 effectively reversed the inhibitory effects of sirt1 AS on 10 ng/ml TGF-β1 induced EMT, as evidenced by the increased protein levels of SMA (Figure 4C) and collagen1 (Figure 4D), as well as the decreased protein levels of E-cadherin (Figure 4E). Moreover, immunofluorescent double staining validated that down-regulation of sirt1 could abolish the inhibitory effects of sirt1 AS on EMT in lung tissues (Figure 4F). Inhibition of sirt1 in BLM-induced mice could reverse the anti-fibrosis effects of sirt1 AS, as evidenced by the H&E and Masson staining (Figure 4G). These results strongly suggested that Sirt1 is involved in sirt1 AS regulated fibrogenesis in IPF.

### ASV inhibited fibroblast activation via upregulating sirt1 AS, *in vitro*

**Figure 5:**
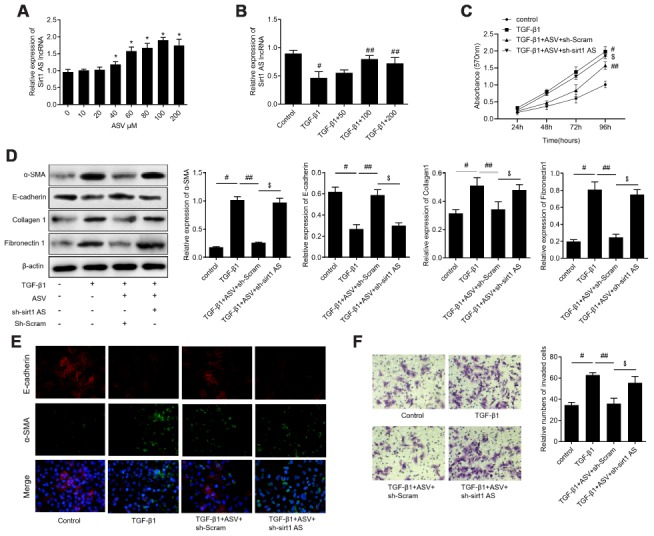
**Knockdown of lncRNA sirt1 antisense (AS) reverses the protective effects of astragaloside IV (ASV) on TGF-β1-induced fibrogenesis.** (**A**) Sirt1 AS expression in RLE-6TN cells treated with various concentration of ASV for 48 h, as indicated. * P<0.05. (**B**) Sirt1 AS expression in TGF-β1 treated RLE-6TN cells upon ASV treatment. # P<0.05 vs. control; ## P<0.05 vs. TGF-β1. (**C**) CCK-8 assay was used to detect the cell viability of RLE-6TN cells treated with 10 ng/ml TGF-β1, TGF-β1+100μM ASV, TGF-β1+ASV+sh-Scramb, TGF-β1+ASV+sh-sirt1 AS. (**D**) Western blotting analysis and quantitative analyses of EMT related markers α-SMA, E-cadherin, collagen1 and fibronectin1 in RLE-6TN cells treated with TGF-β1, TGF-β1+ASV, TGF-β1+ASV+sh-Scramb, TGF-β1+ASV+sh-sirt1 AS. (**E**) Immunofluorescence staining showing the overlap of α-SMA and E-cadherin in RLE-6TN cells with indicated treatment. (**F**) Transwell assay was performed to investigate the migration ability of RLE-6TN cells with indicated treatment. # P<0.05 vs. control group; ## P<0.05 vs. TGF-β1; $ P<0.05 vs. TGF-β1+ASV+sh-Scramb.

We previously reported that ASV has inhibitory effects on TGF-β1-induced EMT in IPF, we then investigate whether sirt1 AS is involved in the function of ASV on IPF. We examined the effects of ASV on sirt1 AS expression and found that 40 μg/ml ASV started to up-regulated sirt1 AS expression and 100 μg/ml ASV influenced sirt1 AS expression at most (Figure 5A). Thereafter, we investigate the effects of ASV on sirt1 AS expression upon TGF-β1 treatment and found that 100 μg/ml ASV could dramatically abolished 10 ng/ml TGF-β1-indcued sirt1 AS inhibition (Figure 5B). As illustrated in Figure 5C, ASV significantly inhibit TGF-β1-indcued cell proliferation of RLE-6TN cells, whereas knockdown of sirt1 AS reversed the inhibitory effects of ASV on cell viability. Besides, by using western blot analysis, ASV was validate to inhibit TGF-β1-indcued EMT of RLE-6TN cells in this study. However, sirt1 AS silencing re-induced the EMT of RLE-6TN cells with ASV plus TGF-β1 treatment (Figure 5D). Immunofluorescence further showed that sirt1 AS silencing could attenuate the changes of α-SMA and E-cadherin caused by ASV treatment (Figure 5E). In addition, Transwell analysis revealed that the ASV inhibited the migratory ability of RLE-6TN cells, which was partly abrogated by down-regulating of sirt1 AS (Figure 5F). These results suggested that ASV may exert its anti-fibrosis at least partly, by regulating sirt1 AS.

### ASV attenuates pulmonary fibrosis through regulating sirt1 AS/sirt1/ foxo3 axis

**Figure 6:**
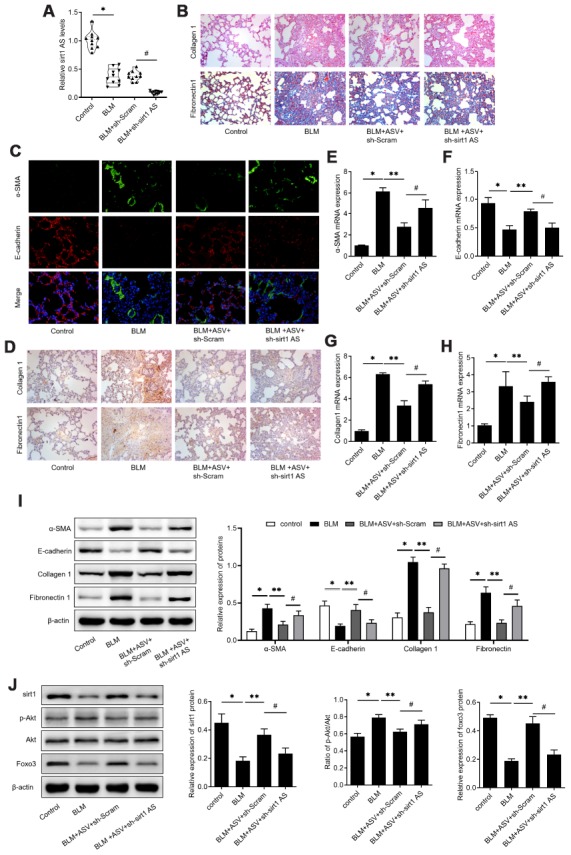
**Astragaloside IV (ASV) alleviates IPF through regulating sirt1 AS/sirt1/ foxo3 axis.** (**A**) Sirt1 AS expression in the pulmonary tissues from control mice, mice treated with bleomycin (BLM), BLM+ adenovirus shRNA against sirt1 AS (ad-sh-sirt1 AS) or BLM+ adenovirus negative shRNA (n=10 per group). (**B**) H&E and Masson’s trichrome staining showed pulmonary injury and collagen deposition of pulmonary tissues in mice treated with bleomycin (BLM), BLM+ adenovirus shRNA against sirt1 AS (ad-sh-sirt1 AS) or BLM+ adenovirus negative shRNA. (**C**) Immunofluorescence staining showing the overlap of E-cadherin (red) and a-SMA (green) in pulmonary tissues from indicated groups. (**D**) Immunohistochemistry analysis indicated the expression of levels of collagen 1 and fibronectin 1 in pulmonary tissues from indicated groups. (**E**–**H**) QPCR analysis demonstrated the relative expression of epithelial-mesenchymal transition (EMT)-related markers, α-SMA, E-cadherin, Collagen 1 and Fibronectin1 mRNA in indicated groups (n=10 per group). (**I**) Western blot assay and quantitative analyses of EMT-related markers, α-SMA, E-cadherin, Collagen 1 and Fibronectin1 protein in indicated groups (n=10 per group). (**J**) Western blot assay and quantitative analyses of sirt1, phosphorylated Akt, Akt and Foxo3 proteins in pulmonary tissues from indicated groups. * P<0.05 vs. control group; ** P<0.05 vs. BLM group; # P<0.05 vs. BLM+ASV+sh-Scramb group.

To validate the implication of sirt1 AS in the anti-fibrosis role of ASV on IPF, a loss-of-function approach was employed. Mice treated with sh-sirt1 AS showed significant decrease in sirt1 AS expression when compared with those treated with sh-Scram (Figure 6A). Knockdown of sirt1 AS at least partly reversed ASV alleviated pulmonary fibrosis, as evidenced by H&E and Masson trichrome staining (Figure 6B). Besides, immunofluorescent double labelled staining of pulmonary tissues showed that suppression of sirt1 AS could abolish ASV-induced α-SMA suppression and E-cadherin upregulation (Figure 6C). The results of immunohistochemistry also revealed that knockdown of sirt1 AS abrogated ASV-induced collagen1 and fibronectin1 upregulation (Figure 6D). Accordingly, after knockdown of sirt1 AS, the expression of α-SMA, collagen1 and fibronectin1 mRNA were increased, while E-cadherin mRNA was decreased (Figure 6E–6H). In addition, western blot analysis revealed that ASV treatment significantly reduced BLM-induced α-SMA, collagen1 and fibronectin1 protein expression, and increased E-cadherin protein expression. After knockdown of sirt1 AS, the expression of α-SMA, collagen1 and fibronectin1 mRNA were increased, while E-cadherin mRNA was decreased (Figure 6I). As we previously demonstrated that ASV inhibited Akt-mediated Foxo3 down-regulation to reverse EMT in IPF [19]. Here, we found that knockdown of sirt1 AS not only reduced ASV-induced sirt1, but also increased phosphorylated Akt to decrease Foxo3 expression in BLM treated pulmonary tissues (Figure 6J). These results indicated that sirt1 AS/sirt1/ foxo3 axis was involved in the anti-fibrosis function of ASV in IPF.

## DISCUSSION

In the present study, we characterized that sirt1 AS inhibited TGF-β1-induecd EMT of alveolar epithelial cells in IPF. sirt1 AS was a negative regulator of EMT and has anti-fibrosis function to alleviate the progression of IPF. The results further revealed that sirt1 AS directly bound sirt1 mRNA to increase the stability of its mRNA, therefore to upregulate sirt1 expression; Sirt1 AS suppressed EMT of alveolar epithelial cells in a sirt1-dependent way. Above data suggested that manipulating sirt1 AS represents promising therapeutic strategies for IPF. Moreover, our results indicated the changes in sirt1 AS expression induced by ASV and sirt1 AS silencing could abolish the protective effects of ASV on IPF. These findings demonstrated a new pathway from ASV to sirt1 AS in regulating EMT for the treatment of IPF.

Many studies have demonstrated that antisense lncRNAs participates in the process of organ fibrosis [22–24]. However, to date, limited study focused on studying the potential role of antisense lncRNAs in IPF. A recent study by Du et al. identified that lncRNA cyclin dependent kinase inhibitor-2B-antisense RNA 1 decreased significantly in patients with IPF compared with healthy controls. It may activate the p53-signaling pathway to promote lung cancer formation [23]. More importantly, we previously found that lncRNA ZEB1-AS1 was upregulated in BLM-treated pulmonary tissues and TGF-β1-induced EMT RLE-6TN cells [14]. Herein, we extended the role of antisense lncRNA into IPF with the finding that Sirt1 AS could significantly regulate TGF-β1-meidated EMT in vitro and in vivo. We found no alteration of ZEB1-AS1 expression in RLE-6TN cells upon ASV treatment (data not shown). Subsequent experiments found sirt1 AS significantly attenuated BLM-mediated IPF. Then we investigated the potential mechanisms underlying the anti-fibrosis function of sirt1 AS.

NATs alter the recruitment of members of the spliceosome to sense transcript by forming RNA-RNA duplexes with the sense transcript [25]. It has been recently shown that several NATs such as GABPB1 antisense, Forkhead box protein C2 (FOXC2) antisense, and keratin 7 antisense, interact with the sense target genes GABPB1, FOXC2, and keratin 7, respectively [26–28], to exert their functions. Sirt1 AS is fully overlapping with the 3’-UTR of sirt1 mRNA and sirt1 AS was reported to be more stable than sirt1 mRNA [17]. Previous studies reported that Sirt1 AS facilitated cell proliferation by binding the 3’-UTR of sirt1 mRNA [29]. Sirt1 AS was mostly located in the cytoplasm of RLE-6TN cells, suggesting it regulated mRNA stability at the epigenetic level. Consistently, the present study validated sirt1 AS directly upregulated sirt1 mRNA and protein expression in alveolar epithelial cells. Moreover, the antisense lncRNA/mRNA duplex may cover miRNA binding sites to prohibit the biological function of miRNAs. Researchers have shown that the miR-34a [18] and miR-22 [30] target sequence on the sirt1 mRNA 3’-UTR overlaps with the corresponding region of Sirt1 AS on the Sirt1 mRNA. However, in the current study, we did not further investigate the potential miRNAs work in the regulation of sirt1 AS/sirt1 axis. Further studies, such as miRNAs sequencing for cells with up or down-regulation of sirt1 AS, are need to identified the exact miRNAs involved in this regulation. Activation of sirt1 has been validated to regulate EMT of pulmonary fibrosis [21, 31]. Here, we found inhibition of sirt1 significantly reversed the anti-fibrosis of sirt1 AS. These results collectively indicated that sirt1 AS directly upregulated sirt1 expression, thereby to inhibit the progress of EMT in IPF.

Astragaloside IV (ASV) is one of the active ingredients of *Astragalus membranaceus* and reported to have protective effects on IPF [32, 33]. Reportedly, ASV treatment down-regulated the expression of lncRNA growth arrest specific 5 in cardiomyocytes to suppress apoptosis [34]. ASV also increased lncRNA H19 expression to attenuate autophagy and mineralization of smooth muscle cells [35]. These findings suggested that the cellular responses to ASV involved lncRNAs. While no data suggest its action related to lnRNAs regulation in IPF. Here, we firstly showed that ASV treatment up-regulated sirt1 AS expression in RLE-6TN cells and down-regulation of sirt1 AS impaired the suppressive effect of ASV on TGF-β1-induced EMT and BLM-induced pulmonary fibrosis. Furthermore, we found that inhibition of sirt1 AS also regulated phosphorylated Akt and Forkhead box o3 (Foxo3) expression induced by ASV. Overexpression of Foxo3a in IPF fibroblasts could suppress the proliferative ability of fibroblasts to attenuated IPF [36]. And we have previously demonstrated that ASV inhibited fibrogenesis by regulating Akt/foxo3 signaling pathway [19]. This study further revealed that ASV influenced Akt/foxo3 pathway by upregulating sirt1 AS/sirt1 axis. Taken together, sirt1 AS was critical for ASV-mediated inhibition of IPF progression. Targeting of sirt1 AS by ASV has opened a new avenue for IPF treatment.

Our study has certain limitations. First, we manipulated the expression of sirt1 AS in mice by intratracheal injection of virus vectors. Gene editing, such as CRISP system. may also be the good approaches. Second, although we identified that sirt1 AS alleviated IPF progression by upregulating sirt1, do other regulators, such as miRNAs, transcription factors and splicing factors, participate in the binding process between sirt1 AS and sirt1 mRNA remain unknown. These questions need to be addressed in future studies. In sum, the present study demonstrated that sirt1 AS attenuated IPF through transcriptionally activating sirt1. In addition, our results showed that ASV/lncRNA sirt1 AS/sirt1/Akt/foxo3 signaling pathway participates in modulating TGE-β1 induced EMT and thus alleviated BLM-induced pulmonary fibrosis, providing a novel molecular basis for the application of ASV in the therapy of IPF.

## MATERIALS AND METHODS

### Animal model and treatment

C57Bl/6J mice were obtained from Shanghai SLAC Laboratory Animal Co., Ltd. and maintained in a 12-hour light-dark cycle and all experimental process were approved by the Ethics Committee of Affiliated Hospital of Shandong University of Traditional Chinese Medicine. The mice of IPF model was achieved by intratracheally injecting with bleomycin (BLM, 1.5 mg/kg, Nippon Kayaku, Japan) dissolved in a total of 50 microliter sterile saline; the control group were instead with equal amounts of sterile saline. After the administration of BLM for three days, adenovirus vectors named adenovirus-control (ad-NC), ad-sirt1 AS, specific short hairpin RNA for sirt1 AS (sh-sirt1 AS) or sh-NC were intratracheally injected into the mouse pulmonary tissues, as previously described [14]. ASV (20 mg/kg; Sigma) treatment was started at day 15 and treated daily for 14 days by gavage, as previously described [19]. All mice were euthanized on day 28^th^ and pulmonary tissue sections were collected for further studies.

### Cell culture and treatment

RLE-6TN alveolar epithelial cells (ATCC, Rockville, Maryland, USA) were cultured in F12 medium (Biowset, Riverside, MO, USA) with 10% Fetal Bovine Serum (FBS, Gibco, MD, USA), penicillin (100 U/mL) and streptomycin (100 μg/mL) in a humidified incubator at 37 °C with 5 % CO_2_. As we previously described [14], treatment with 10 ng/ml TGF-β1 (R&D Systems, Minneapolis, MN) for various time was served as the cell model of EMT. Cells were also treated with various concentration (10-200 μM) of ASV (Xiya Reagent, Shandong, China) for 48 h, if mentioned.

### Adenovirus and small RNA transfection

The adenovirus overexpression sirt1 AS (ad-sirt1 AS) were synthesized by Shanghai Genechem Co.,Ltd. Ad-sirt1 AS was used to infect with RLE-6TN cells as previously described [37]. Lentiviral expression vectors of pLVX-sh-sirt1 AS were constructed by Genechem Co.,Ltd. Sirt1 si-RNA was designed and synthesized by Ribo Bio Technology (Guangzhou, China). The sequences of the above oligonucleotides were listed in Supplementary Table 1. For the transfection in vitro, cells were transfected with the adenovirus at multiplicities of infection (MOI) of 20 for 3 h before medium change. For sh-RNA/si-RNA transfection, about 2×10^5^ RLE-6TN cells were plated in six-well culture dishes prior to small RNA transfection. On the following day, cells were washed with serum-free medium and starved for 6 h. Subsequently, sh-RNA/si-RNA and Lipofectamine 2000 (Invitrogen) were separately mixed with Opti-MEM I Reduced Serum Medium for 5 min and the two mixtures were incubated at room temperature for 20 min. The Lipofectamine sh-RNA/si-RNA mixture was added to the cells and incubated at 37 °C for 6 h and fresh medium containing 10% FBS was added. The cells were maintained in culture until subsequent experiments. For the transfection in vivo, mice were intratracheally delivered with adenovirus control, Ad-sirt1 AS + Ad-si-NC, or Ad- sirt1 AS + Ad-si-sirt1 on the third day after BLM treatment.

### Pathological staining

The isolated lung tissues were fixed in 4% paraformaldehyde, decalcified, dehydrated, cleared with dimethylbenzene and embedded in paraffin. The 5 μm sections were stained with either Hematoxylin and Eosin or Masson's trichrome staining using standard procedures. Immunohistochemistry was performed to measure the expression of collagen1 or fibronectin1. Sections were incubated with 3% hydrogen peroxide to block endogenous peroxidase activity. Following incubation with the primary antibody at 4°C overnight, the slides were treated with DAB (Beyotime, Jiangsu, China) to visualize the immunoreactivity. The images were obtained using the Leica DM3000 microscope (Leica Company, Germany).

### Immunofluorescence staining

After treatment, cells were fixed with 4% formaldehyde for 5 mins. Following incubation with anti-α-SMA (ab5694, Abcam) or E-cadherin (ab40772, Abcam) antibodies and fluorescent-labeled secondary antibodies (Alexa Fluor® 488 or Alexa Fluor® 594), DAPI (Life Technologies Corporation) was used to stain nuclear counter. Images were captured by a Nikon Eclipse 800 epifluorescence microscope with the appropriate filters.

### Fluorescence in situ hybridization (FISH)

RLE-6TN cells were fixed with 4% paraformaldehyde and then incubated with 0.2mol/L HCl followed by proteinase K treatment. After pre-hybridization, cells were incubated with pre-hybrid buffer with 0.1 μg FITC-labeled sirt1 AS probe at 37 °C overnight and washed with 2 × SSC (37 °C, 10 min) and 1 × SSC (37 °C, 5 min, 2 times) in order. Cells were then incubated with FITC-labeled streptavidin (1:400) at room temperature, washed with PBS and DAPI (sigma) was used for nuclear staining.

### Real-time quantitative PCR

Real-time quantitative PCR (qPCR) was performed to detect the expression of sirt1 AS, and fibrogenesis-related genes. GAPDH was included as an endogenous control. In briefly, total RNA was extracted using TRIzol (Invitrogen). Concentration of the RNA was assessed with NanoDrop-2000 Spectrophotometer (NanoDrop Technologies, Germany), then reverse transcription using a PrimeScript reagent Kit With gDNA Eraser (Takara). The primers of target genes were synthesized by Shanghai bioengineering Co.Ltd. (Supplementary Table 2). QPCR was performed with a SYBR Green PCR kit (TaKaRa) and the expressions of target genes were calculated using the 2^−ΔΔCt^ method.

### Nuclear-cytoplasm separation

RLE-6TN cells were treated according to the manufacturer’s instructions (Thermo Fisher Scientific) to obtain separate nuclear and cytoplasm parts. RNA extraction and qPCR were then conducted for nuclear and cytoplasm parts, respectively.

### Half-life of sirt1 mRNA

For mRNA stability measurements, RLE-6TN cells were treated with 2 μg/ml actinomycin D (MedChemExpress, Shanghai, China), which suppresses transcription. Total RNA was extracted at indicated time point (0, 1, 2, 4, 6, 8, and 10 h) to determine the residual mRNAs by using qPCR analysis. β-actin was served as the internal control.

### Western blot

Cultured cells and tissues were lysed with RIPA buffer (Beibo, China) with phosphatase inhibitor cocktail (Sigma, St. Louis, MO, USA) and then protein for further western blot following the protocol as describe previously [38]. The antibodies were used in present study as following: α-SMA (ab5694, Abcam), E-cadherin (ab40772, Abcam), Collagen1 (ab34710, Abcam), Fibronectin1 (FN1, ab2413, Abcam), sirt1 (ab110304, Abcam), Foxo3 (ab23683, Abcam), p-Akt (phospho Ser473, ab81283, Abcam), Akt (ab8805, Abcam), and β-actin (sc-70319, Santa Cruz).

### Cell proliferation and migration

RLE-6TN cells with a cell density of 5,000 cells/well were seeded in 96-well plates. After incubating for 24 h, the cells were treated with varying interventions and then the cells were incubated with CCK-8 solution (Dojindo, Kumanoto, Japan) for 2 h. A Microplate Reader (Model 680, Bio-Rad, Hercules, CA, USA) was used to measure the absorbance at 450 nm. After transfection, cells were trypsinized, harvested, and resuspended in serum-free F12 medium and seeded in the upper chamber of a Transwell chamber at a cell density of 1 × 10^5^/ml. The lower chamber contained 500 μl of F12 medium containing 10% FBS. After 24 h’ culture, the cells that migrated to the other side of the upper chamber were fixed with 4% paraformaldehyde and subjected to crystal violet staining. The number of transmembrane cells was artificially counted.

### Statistical analysis

Data are expressed as the mean ± SD. Student’s t-test or ANOVA was used to compare the means of two or three groups. All statistical analyses were conducted using GraphPad Prism v8 (GraphPad Prism, Inc., La Jolla, CA, USA). The significance level was set at P<0.05.

## Supplementary Material

Supplementary Tables
