# Supplementary material for: Sirt1 antisense long non-coding RNA attenuates pulmonary fibrosis through sirt1-mediated epithelial-mesenchymal transition
Source: Aging (Albany NY). 2020 Mar 6;12(5):4322–36. doi: 10.18632/aging.102882 (PMC7093192; doi:10.18632/aging.102882)
Supplement: Supplementary Tables [file aging-12-102882-s001..pdf]

SUPPLEMENTARY MATERIALS

Supplementary Tables

Supplementary Table 1. The forward and reverse primers for real-time PCR.

Genes		5’-3’ primer sequence
overexpression vectors (mouse)		
Sirt1 antisense lncRNA	forward	ATATTAAGCCGGTCGGTGGATTCTCCCGATTGT
	reverse	ATCATATAGGCGCCTGGGAAATTGCGTCA
Negative control	forward	ATACTCGAGCAAATGAGAGAGTATGGTGTGGT
	reverse	ATAGAATTCCCAAACTTGCCCATAACCATAGG
Sirt1 sh-RNA	forward	TTGGCACCGATCCTCGAAC
Sh-Scramble	forward	GCTTGTTCAGAGAACAATTGC
Sirt1 si-RNA	forward	ACUUUGCUGUAACCCUGUA
Sirt1 si-NC	forward	ATGGGTGTGCTAGCATGTT

Supplementary Table 2. The forward and reverse primers for real-time PCR.

Genes		5’-3’ primer sequence
**Sirt1 AS**	forward	AATCCAGTCATTAAACGGTCTACAA
	reverse	TAGGACCATTACTGCCAGAGG A
**Sirt1**	forward	TTGGCACCGATCCTCGAAC
	reverse	CCCAGCTCCAGTCAGAACTAT
**α-SMA**	forward	GTGTTGCCCCTGAAGAGCAT
	reverse	GCTGGGACATTGAAAGTCTCA
**E-cadherin**	forward	GCTTTGTTAAGGCTGGTGAT
	reverse	TGGAGAAGATTGGCTAGGTT
**Col1A1**	forward	ACACGCTCAACGTCAGCAT
	reverse	GCTCGTCCAGCTCTGGATAT
**Fibronectin1**	forward	TGCCAACCTTTACAGACCTATC
	reverse	AGCACGAGTCATCCGTAGGT
**GAPDH**	forward	TGCTGAGTATGTCGTGGAGTCT
	reverse	ATGCATTGCTGACAATCTTGAG
